# Restoration of tendon repair microenvironment by grapefruit exosome-loaded microneedle system for tendinopathy therapy

**DOI:** 10.3389/fbioe.2025.1615650

**Published:** 2025-07-28

**Authors:** Yuan Zhang, Ruiyang Zhang, Ti Zhang, Yuhao Mu, Talante Juma, Xu Li, Hao Li, Quanyi Guo, Yongping Cao

**Affiliations:** ^1^Department of Orthopedics, Peking University First Hospital, Beijing, China; ^2^Key Laboratory for Regenerative Medicine of the Ministry of Education of China, School of Biomedical Sciences, Faculty of Medicine, The Chinese University of Hong Kong, Shatin, Hong Kong SAR, China; ^3^Beijing Key Lab of Regenerative Medicine in Orthopedics, Key Laboratory of Musculoskeletal Trauma & War Injuries PLA, Institute of Orthopedics, The First Medical Center, Chinese PLA General Hospital, Beijing, China; ^4^Department of Orthopedics, Peking University Third Hospital, Beijing, China; ^5^Beijing Jishuitan Hospital, Capital Medical University, Beijing, China

**Keywords:** tendinopathy, grapefruit-derived exosome, microneedles, oxidative stress, macrophage polarization

## Abstract

Tendinitis repair remains challenging due to the limited self-renewal capacity of tenocytes and persistent inflammatory microenvironment. Conventional therapies remain limited by systemic drug toxicity and fail to coordinate immunomodulation with matrix remodeling. Plant-derived extracellular vesicles have demonstrated tissue repair potential owing to their unique bioactive components and exceptional cross-species compatibility. Nevertheless, their therapeutic role in tendon matrix regeneration remains underexplored. Here, we developed a grapefruit-derived exosome-loaded microneedle patch (MN@GF-Exos) to synergistically restored tendon structure and functions. Grapefruit-derived exosomes (GF-Exos) were loaded into dissolvable hyaluronic acid microneedles (MNs) for sustained release. GF-Exos reversed oxidative stress in tenocytes, enhancing cellular proliferation and migration, restoring collagen I synthesis, and polarizing macrophages toward M2-repair phenotypes. Transcriptomics revealed GF-Exos modulated cytokine-cytokine receptor interactions, suppressing inflammation-related pathways and activating ECM organization genes. In collagenase-induced tendinopathy mice, MN@GF-Exos enhanced gait recovery and extracellular matrix remodeling. Histology confirmed reduced fibrosis without ectopic ossification. Systemic safety was validated by unchanged organ histology and within-normal-limits serum biomarkers. This dual-functional system leverages plant exosomes’ multi-component synergy and MN’s spatiotemporal control, offering a translatable strategy for chronic tendon regeneration.

## 1 Introduction

Tendinitis, a clinically prevalent condition resulting from excessive mechanical loading, is characterized by degenerative changes in tendon collagen fibers (Milla et al., 2021). Its pathological hallmarks include dysregulation of extracellular matrix (ECM) homeostasis, exacerbated inflammatory infiltration, and disorganized collagen architecture (Milla et al., 2021). Tendinopathy affects approximately 6% of the general population and 30% of runners in the world (Kwan et al., 2023; Loiacono et al., 2019; Traweger et al., 2025). Current clinical interventions primarily aim to alleviate pain symptoms using topical or oral anti-inflammatory drugs, while severe tendon injuries are addressed through surgical repair (Milla et al., 2021; Li et al., 2025). However, these treatment approaches are constrained by systemic administration-related issues such as gastrointestinal toxicity, low local drug penetration efficiency, and poor targeting, which significantly limit their capacity to achieve sustained tissue-level repair. (Moroz et al., 2016). Engineering innovative therapeutic systems with effective delivery and local microenvironment modulation capabilities has emerged as a pivotal challenge in tendon regenerative medicine, particularly for addressing degenerative pathologies characterized by chronic inflammation and ECM disorganization.

In recent years, plant-derived extracellular vesicles (PELNs) have shown potential beyond traditional drug carriers due to their unique bioactive components (such as lipids, miRNAs, and natural small molecules) and demonstrable cross-species compatibility (Mu et al., 2023; Zhao et al., 2024; Xu et al., 2024; Feng et al., 2023). Grapefruit-derived exosomes (GF-Exos) as an important subclass of PELNs have been proven to exert significant anti-inflammatory and antioxidant effects in colitis, psoriasis, and atopic dermatitis models by delivering active molecules such as naringenin and flavonoids (Huang et al., 2023; Wang et al., 2014). Moreover, GF-Exos have also been shown to have the ability to promote cell proliferation and migration, making them a potential cell-free wound healing therapeutic agent (Savcı et al., 2021). However, the therapeutic potential of GF-Exos in tendinopathy has not been studied yet.

In the field of tissue engineering and regenerative medicine, microneedles (MNs) are commonly used as scaffolds to regulate the microenvironment at the site of injury by releasing inflammation-suppressing drugs, transmitting signaling factors, and growth factors (Zhang et al., 2023). MNs penetrate the epidermal barrier through a micrometer-sized needle array and can directly deliver drugs to the dermis or surrounding tissues. It is characterized by non-invasiveness, high penetration efficiency, and local sustained-release advantages (Chakraborty et al., 2024). Compared to traditional injections, MN delivery can significantly reduce systemic exposure risks and prolong the duration of drug action at the injury site, especially suitable for chronic tendon repair processes that require long-term intervention (Cammarano et al., 2024).

**FIGURE 1:**
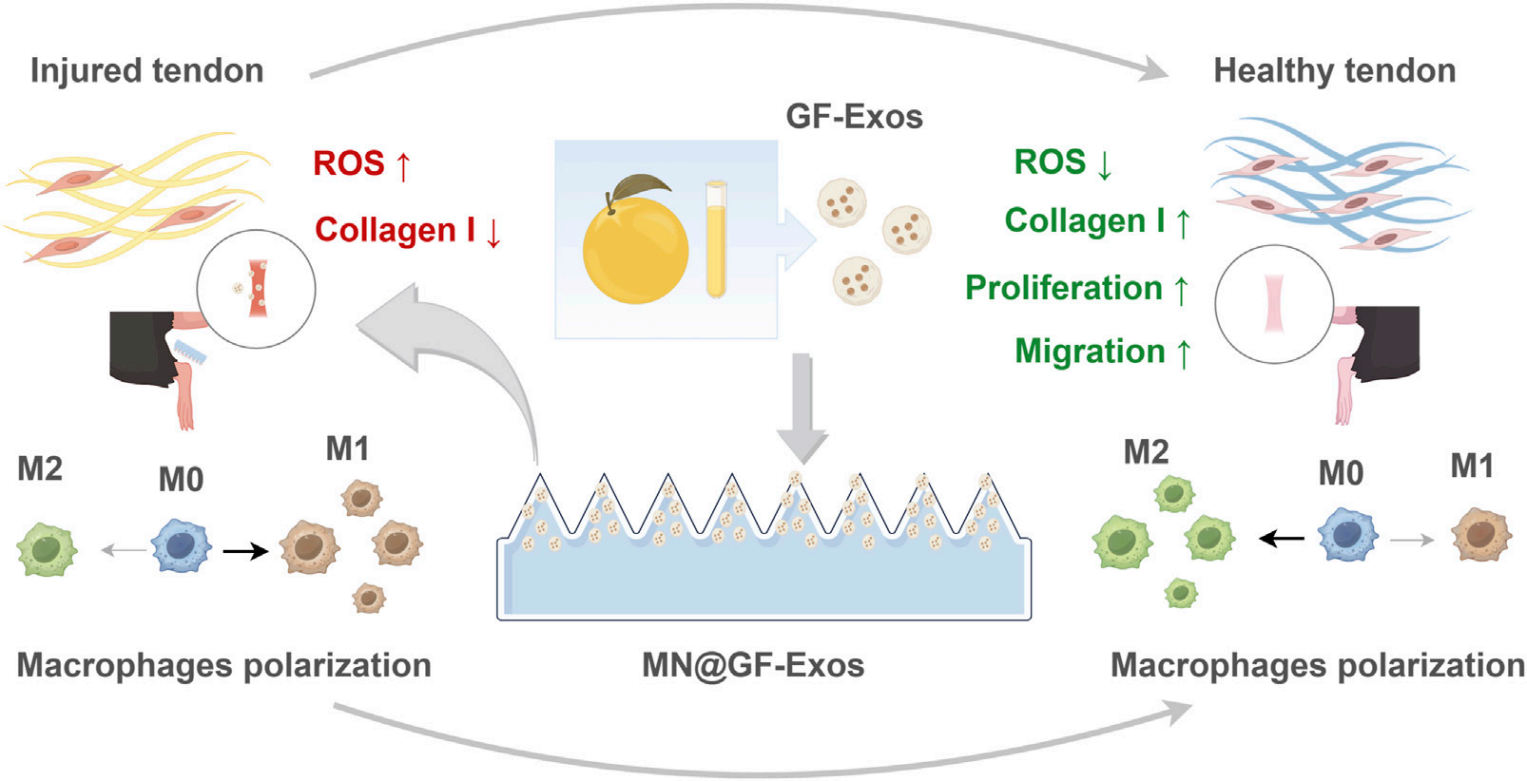
Schematic illustrating MN@GF-Exos-mediated therapeutic mechanisms against tendinopathy through coordinated modulation of tenocyte proliferation/migration enhancement, oxidative stress suppression, collagen I synthesis restoration, and M2 macrophage polarization (By Figdraw).

This study innovatively loaded GF-Exos onto a soluble microneedle patch (MN@GF-Exos), aiming to construct a targeted therapeutic system that combines anti-inflammatory and regenerative effects (Figure 1). *In vitro* experiments confirmed that GF-Exos could reverse the increase in ROS levels and functional inhibition induced by hydrogen peroxide in tendon cells (i.e., restoration of type I collagen synthesis and enhancement of cell migration and proliferation ability). Moreover, GF-Exos demonstrated the ability to modulate macrophage polarization toward the M2 phenotype. Further combined with the spatiotemporal controlled-release characteristics of microneedles, it achieved the repair of tissue structure and restoration of the mechanical function in a mouse model of tendonitis induced by collagenase. This study provided new ideas for the clinical translation of plant-derived vesicles and precise treatment of tendon diseases.

## 2 Materials and methods

### 2.1 Preparation of grapefruit-derived exosomes

Exosomes derived from grapefruit were extracted via sucrose density gradient centrifugation. Firstly, the fresh pulp of grapefruit was homogenized and centrifuged at 3000×g for 20 min to remove residue. Then the supernatant was centrifuged at 10,000×g for 40 min to remove large particles. The supernatant was subjected to ultracentrifugation (150,000×g, 4°C, 2 h) to preliminarily enrich the crude exosome extract. The precipitate was resuspended in PBS and layered onto a pre-prepared sucrose gradient solution (8%, 30% and 45% concentration gradient) for ultracentrifugation (150,000×g, 4°C, 2 h) to achieve high-precision separation of exosomes from other vesicle subpopulations. The target exosomes (GF-Exos) were collected in the 30%–45% density range. The morphology was observed using transmission electron microscopy (JEOL) and the particle size was analyzed.

### 2.2 Preparation and characterization of GelMA hydrogel microneedles loaded with grapefruit-derived exosomes

GelMA 60 solution [10% (w/v)] was mixed with GF-Exos at a ratio of 1:9 (v/v) (final concentration of exosomes 100 μg/mL). The mixed solution was dropped onto the surface of PDMS molds to cover the microneedle array. The molds were vacuum degassed at - 0.1 MPa for 5 min at room temperature, and the residual solution with bubbles was removed. The molds were heated in a 30°C constant temperature oven for 5 h to concentrate the solution by evaporation of water. The heating process was repeated after supplementing the mixed solution. The microneedles were preliminarily solidified by irradiating with 365 nm UV light (intensity 10 mW/cm^2^) for 60 s. The PDMS molds were gently peeled off to obtain the microneedles loaded with exosomes. The integrity of the microneedles was observed under an optical microscope and a scanning electron microscope.

### 2.3 Mechanical strength testing

The mechanical properties of microneedle arrays were evaluated using a universal testing machine (UTM-510, Sansi Testing Equipment Co., Ltd.) equipped with a 100 N load cell. Specimens (10 × 10 mm^2^) were mounted on the stage with cyanoacrylate adhesive and subjected to compression testing under a displacement rate of 1.2 mm/min. Force-displacement curves were recorded and normalized mechanical strength (N/needle) was calculated by dividing the peak force (N) by the total number of needles in the loaded area.

### 2.4 Isolation of primary tendon cells

The Achilles tendon tissue of 8-week-old SD rats was collected and was washed three times with PBS. The tissue was minced into 1 mm^3^ fragments and digested with 0.2% type I collagenase (Sigma, C0130) at 37°C for 45 min. After digestion, the fragments were passed through a 70 μm cell sieve (FALCON, 352,350) and centrifuged (1,000 × g, 5 min) to collect the cell precipitate. The cell suspension was resuspended in DMEM medium containing 10% FBS (Vazyme, F101-01) and 1% penicillin/streptomycin (OriCell, ATPS-10001) and inoculated onto collagen-coated plates. The cells were cultured at 37°C, 5% CO_2_ for 3 days and then passaged to the third generation for subsequent experiments.

### 2.5 Detection of oxidative stress by DCFHDA

P3 generation tendon cells were seeded in 96-well plates at a density of 5 × 10^3^ cells per well. The control group was cultured in conventional medium. The H_2_O_2_ group was treated with 100 μM H_2_O_2_ for 24 h before staining. The exosome group cells were co-cultured with 50 μg/mL grapefruit-derived exosomes for 24 h. The DCFH-DA probe (Beyotime, S0035S) was diluted 1:1,000 in serum-free medium according to the instructions and the final concentration was 5 μM. The medium was discarded, and the diluted probe was added to incubate at 37°C in the dark for 30 min. The cells were washed three times with PBS and the ROS level was detected using a fluorescence microplate reader (Ex/Em = 495/530 nm).

### 2.6 Detection of cell proliferation by EdU

Primary tendon cells were seeded in 24-well plates (2 × 10^4^ cells per well). The cells were treated with 50 μg/mL grapefruit-derived exosomes for 24 h. The cells were Incubate with 50 μM EdU (Beyotime, C0078S) for 2 h. Then, the cells were fixed with 4% paraformaldehyde, and permeabilize with 0.5% Triton X-100. The Click reaction solution was added and the samples were incubated in the dark for 30 min. The nuclei were stained with Hoechst (Beyotime, C0078S) (1:1,000). The EdU positive rate = number of red fluorescent cells/total cell number × 100%.

### 2.7 Immunofluorescence staining

Primary tendon cells were suspended in 24-well plates (2 × 10^4^ cells/well). The cells were treated with 100 μM H_2_O_2_ or 100 μM H_2_O_2_ combined with 50 μg/mL grapefruit-derived exosomes for 24 h. After treatment, cells were fixed with 4% paraformaldehyde for 15 min, permeabilized with 0.1% Triton X-100 for 10 min, and blocked with 5% BSA for 1 h. Subsequently, cells were incubated with anti-COL1A1 antibody (Abcam, ab6308), anti-phospho-samd2/3 antibody (AP0548, ABclonal), anti-COL3A1 antibody (A3795, ABclonal), and anti- ACTA2 antibody (A1011, ABclonal) at 4°C overnight. After washing, Alexa Fluor 488-labeled secondary antibody (1:500) (UElandy, Y6104L) was added and incubated at room temperature for 1 h. Phalloidin-TRITC (1:200) (Solarbio, CA1610) was then applied at room temperature for 40 min, followed by two PBS washes. Finally, DAPI (1:1,000) was incubated in the dark at room temperature for 10 min. Images were captured using a confocal microscope (Zeiss), and fluorescence intensity is quantified with ImageJ.

### 2.8 Scratch migration assay

Primary tendon cells were suspended in 6-well plates until 90% confluence was reached. Scratches were created using a 200 μL pipette tip. The control group was cultured with serum-free medium, while the exosome group was treated with serum-free medium containing 50 μg/mL grapefruit exosomes. Images were captured at fixed positions using an inverted microscope (Nikon) at 0, 12, and 24 h. The migration rate was calculated as: (initial scratch area - remaining area)/initial area × 100%.

### 2.9 Transwell migration assay

Transwell chambers with 8 μm pore diameter (Corning) were placed in 24-well plates. Primary tendon cells (1 × 10^4^ cells/well) were suspended in the upper chamber with serum-free medium. The lower chambers were filled with either serum-free medium or medium containing 50 μg/mL grapefruit exosomes for 24 h. After incubation, cells were fixed with 4% paraformaldehyde and stained with 0.1% crystal violet. The migrating cells were counted in 3 randomly selected fields.

### 2.10 Macrophage polarization assay

Mouse alveolar macrophages were suspended in 24-well plates (2 ×10^4^ cells/well). The cells were treated with 100 ng/mL LPS (Beyotime, ST1470) alone or in combination with 50 μg/mL grapefruit-derived exosomes for 24 h. After treatment, cells were fixed with 4% paraformaldehyde and stained with DAPI. Images were randomly captured under a confocal microscope (Zeiss), and migrating cells were counted in the field.

### 2.11 Exosome uptake assay

Exosomes were labeled with PKH26 dye (Solarbio, D0030) and co-incubated with tendon cells for 6 h. Cells were fixed with 4% paraformaldehyde and stained with DAPI for nuclear visualization. Red fluorescence (Ex/Em = 551/567 nm) indicating exosome localization was observed under a confocal microscope.

### 2.12 RNA-sequencing of tendon cells

Primary tendon cells were categorized into H_2_O_2_ group and H_2_O_2_+MN@GF-Exos groups. The H_2_O_2_ group was treated with 100 μM H_2_O_2_, conducted in triplicate. In contrast, the H_2_O_2_+MN@GF-Exos group cells were co-cultured with 50 μg/mL grapefruit-derived exosomes. Following 24-h incubation, cells were washed twice with PBS, detached using 0.25% trypsin-EDTA, and collected by centrifugation at 300×g for 5 min. Total RNA was extracted using Trizol reagent through a standardized protocol: cell pellets were homogenized in 1 mL Trizol, incubated for 5 min at room temperature, then mixed with 200 μL chloroform. After phase separation by centrifugation (12,000×g, 15 min, 4°C), RNA in the aqueous phase was precipitated with 500 μL isopropanol and washed with 75% ethanol. RNA quality was verified using the NanoDrop™ 2000 spectrophotometer (Thermo Fisher Scientific) and Agilent 2,100 Bioanalyzer system (Agilent Technologies). The RNA library and sequencing was performed commercially by Novogene Co., Ltd (Beijing, China) using the Illumina NovaSeq 6,000 platform. The subsequent bioinformatics analyses were conducted on Novogene’s online platform (NovoMagic), accessed via https://magic-plus.novogene.com/#/. The pipeline included Pearson correlation analysis, differential expression analysis, Gene Ontology (GO), Kyoto Encyclopedia of Genes and Genomes (KEGG) pathway analysis, and Gene Set Enrichment Analysis (GSEA). Differentially expressed genes (DEGs) were identified using DESeq2 with thresholds of |log2 fold change| > 1 and adjusted p-value < 0.05. Default parameters were used unless otherwise specified. The platform’s integrated GO, KEGG and GSEA module was applied for GO, KEGG, and GSEA analysis. Default parameters were applied. Raw sequencing data were deposited in the NCBI SRA under accession number SUB15234643.

### 2.13 Establishment of mouse tendinitis model

To establish the tendinitis model, select 10-week-old male C57BL/6J mice. These mice received two injections of 20 μL of collagenase I (5 mg/mL, Gibco, Cat# 17100017) at the midpoint of the right Achilles tendon 7 days and 4 days before the experiment, respectively. The tendon disease model was confirmed to be successfully constructed through clinical assessment. The mice were randomly divided into three groups: untreated group, empty MN group, and MN@GF-Exos group. Empty MN or MN@GF-Exos were administered after the clinical assessment. All animal trials received approval from the Institutional Animal Care and Use Committee of PLA General Hospital (SCXK No. 2019-0018). Following the application of DiD-labeled exosome-loaded microneedles (GF-Exos MN) to the tendon region of mice, longitudinal fluorescence imaging was performed using a small animal *in vivo* imaging system (*In-Vivo* Xtreme, BRUKER) at postoperative days 3, 5, 7, and 14. The near-infrared fluorescence signals emitted by the DiD tracer (V22887, Invitrogen) were quantitatively monitored at each timepoint to track the retention and distribution dynamics of the delivered exosomes within the tendon tissue.

### 2.14 Functional evaluation and histological assessment

At the fourth week after treatment, gait analysis was performed using the CatWalk XT system (Noldus): the mice were placed on a glass walking platform and at least 3 valid walking sequences were recorded (step speed range 10–30 cm/s). Subsequently, the mice were sacrificed, and tendon calcification or osteophyte formation was evaluated using a small animal X-ray imaging system (Faxitron, 45 kV, 2 s exposure). Tensile mechanical properties of murine tendon specimens were evaluated using uniaxial load-to-failure testing (n = 10 per group). Tendons samples were harvested at week 2 post-treatment and mounted in a materials testing system (5848 MicroTester, Instron) with cryoclamped ends immersed in 37°C PBS to maintain physiological hydration. Following pre-conditioning (10 cycles at 0.1% strain), samples underwent tensile loading at 0.1 mm/s until rupture. Key parameters were recorded.

Tendon samples were collected and fixed in 4% paraformaldehyde overnight, then dehydrated and embedded in paraffin. 5 μm paraffin sections were prepared for Hematoxylin and Eosin (H&E, Hunan Bkmam Holdings Co., Ltd.), Masson’s trichrome staining (Solarbio), and Sirius Red staining (). For immunohistochemistry, sections were blocked at room temperature after antigen retrieval, followed by overnight incubation at 4 °C with anti-I type collagen (14695-1-AP, Proteintech, 1:500) and III type collagen (22734-1-AP, Proteintech, 1:500) antibodies. After DAB staining, the percentage of positive area was calculated. The orientation of collagen fibers was quantified using the Directionality plugin of ImageJ software.

### 2.15 Biological safety evaluation

Blood samples were collected from the mouse heart through puncture or the posterior venous plexus of the eye using non-anticoagulant tubes, with gentle operation to avoid hemolysis. The blood volume was controlled according to body weight (approximately 0.5–1.0 mL/20 g). The blood samples were left to stand at room temperature for 30 min to promote clot formation, then centrifuged at 2000×g at 4°C for 15 min, and the upper pale-yellow serum was aspirated into sterile EP tubes, avoiding touching the blood cell layer. The samples were immediately tested or aliquoted and stored at −80°C (to avoid repeated freezing and thawing). The samples were thawed and the metabolic parameters (ALT, AST, TBIL, DBIL, CREA and UREA) were determined using the automatic biochemical analyzer (BIOBASE, BK280). The parameters included Alanine Aminotransferase (ALT), Aspartate Aminotransferase (AST), Total Bilirubin (TBIL), Direct Bilirubin (DBIL), Creatinine (CREA) and Urea. The entire operation was maintained at a low temperature (4 °C ice box operation), and the data were standardized as the content of target substances per mL of serum (ng/mL or U/mL).

### 2.16 Statistical analysis

All data were presented as the mean ± Standard Error of the Mean (SEM). Statistical significance was determined by one-way ANOVA with Fisher’s LSD test. (GraphPad Prism 9). P < 0.05 was considered significant.

## 3 Results

### 3.1 Extraction and characterization of exosomes derived from grapefruit source

**FIGURE 2:**
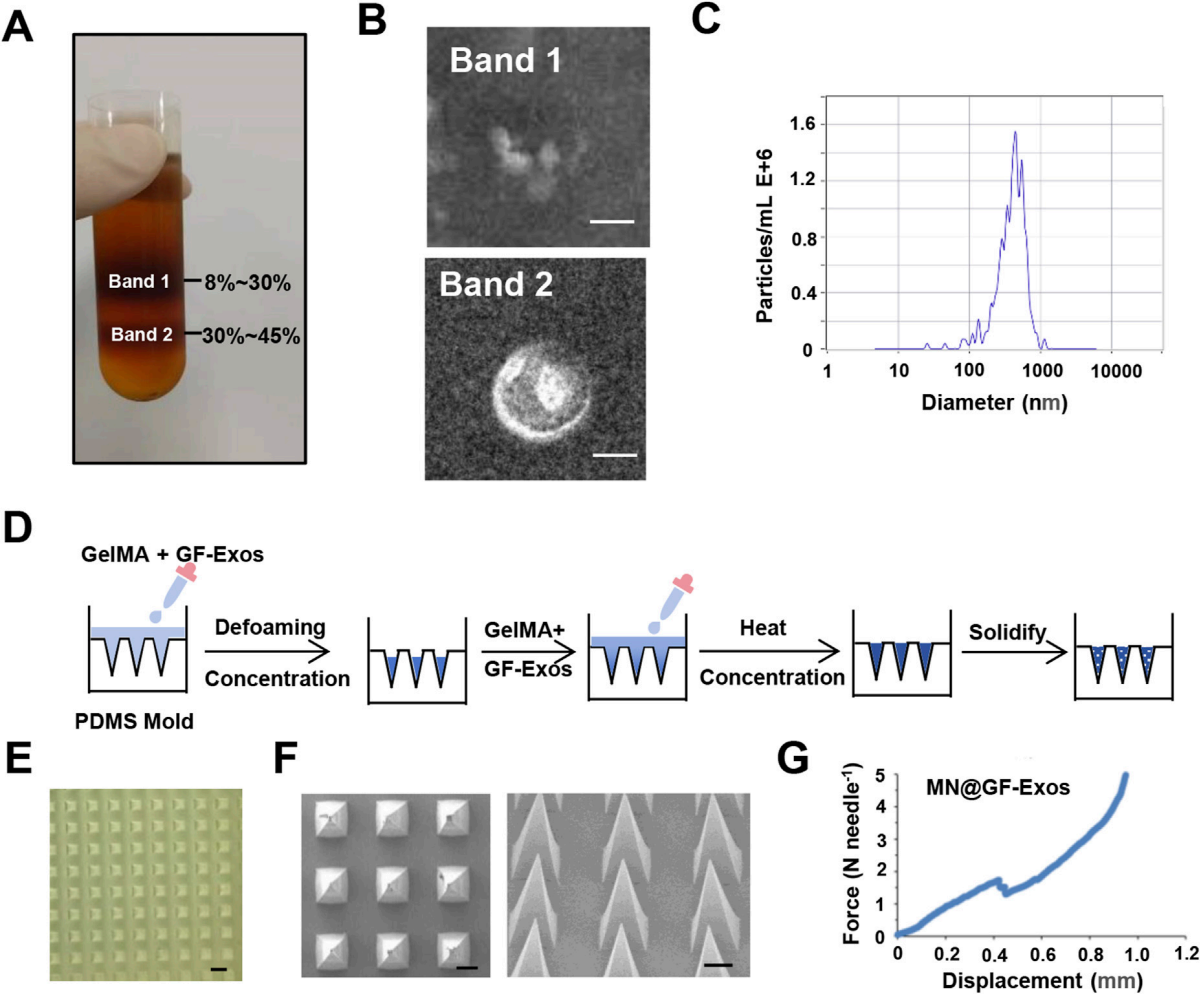
Characterization of GF-Exos and Microneedles. **(A)** Representing images of sucrose density gradient centrifugation. **(B)** Transmission electron microscopy (TEM) images depicting exosomes from Band 1 and Band 2. (Scale bar = 200 µm) **(C)** Particle size of GF-Exos determined by Nanoparticle tracking analysis (NTA). **(D)** Diagrammatic representation of the synthesis pathway for MN@GF-Exos. **(E)** Optical Micrographs of MN@GF-Exos. (Scale bar = 500 µm) **(F)** SEM Micrographs of MN@GF-Exos. (Scale bar = 200 µm) **(G)** Mechanical Characterization of MN@GF-Exos.

Exosomes derived from grapefruit source were extracted by density gradient centrifugation (Figure 2A). Two bands were formed in the sucrose gradient solution, where band 1 appeared as debris under the electron microscope and had no complete vesicular structure, while band 2 could be found to contain vesicular substances (Figure 2B). Nanoparticle tracking analysis (NTA) showed that the median particle size was approximately 400 nm (Figure 2C). To evaluate the thermal stability of GF-Exos during microneedle fabrication, the exosomes were subjected to two cycles of thermal stress (30°C for 5 h per cycle) simulating the MN@GF-Exos manufacturing protocol. Subsequent characterization through transmission electron microscopy (TEM) and nanoparticle tracking analysis (NTA) demonstrated preserved vesicle integrity, while the median particle diameter remained stable at 400 nm (Supplementary Figure S1A; Supplementary Figure S1B).

### 3.2 Preparation and characterization of micro needles loaded with exosomes

Micro needles were prepared as shown in Figure 2D. Optical microscopy and scanning electron microscope (SEM) imaging demonstrated that the micro needle array maintained uniform tetrahedral shapes, with uniform and very sharp tips (Figures 2E,F), meeting the basic geometric standards required for tissue penetration. The heights of these micro needles were 860 μm, and the base widths were 360*360 μm. Mechanical strength of the micro needles was tested *in vitro* (Figure 2G). The sudden drop point in the force-displacement curves referred to the microneedle mechanical failure. The fracture force of MN@GF-Exos was around 1.7 N (Figure 2G), which was higher than those needed for skin penetration (0.1–3N) (Davis et al., 2004), indicating that the mechanical strength of the micro needles prepared in this study is sufficient to puncture the skin.

### 3.3 GF-Exos-mediated enhancement of tendon cell proliferation and migration

A CCK-8 assay was performed to determine the maximum non-toxic concentration of GF-Exos on tendon cells and 50 μg/mL was the highest dose that maintained >90% cellular viability and showed no statistically significant differences in viability compared to that of untreated controls (Supplementary Figure S2). This concentration was used in the following experiments.

**FIGURE 3:**
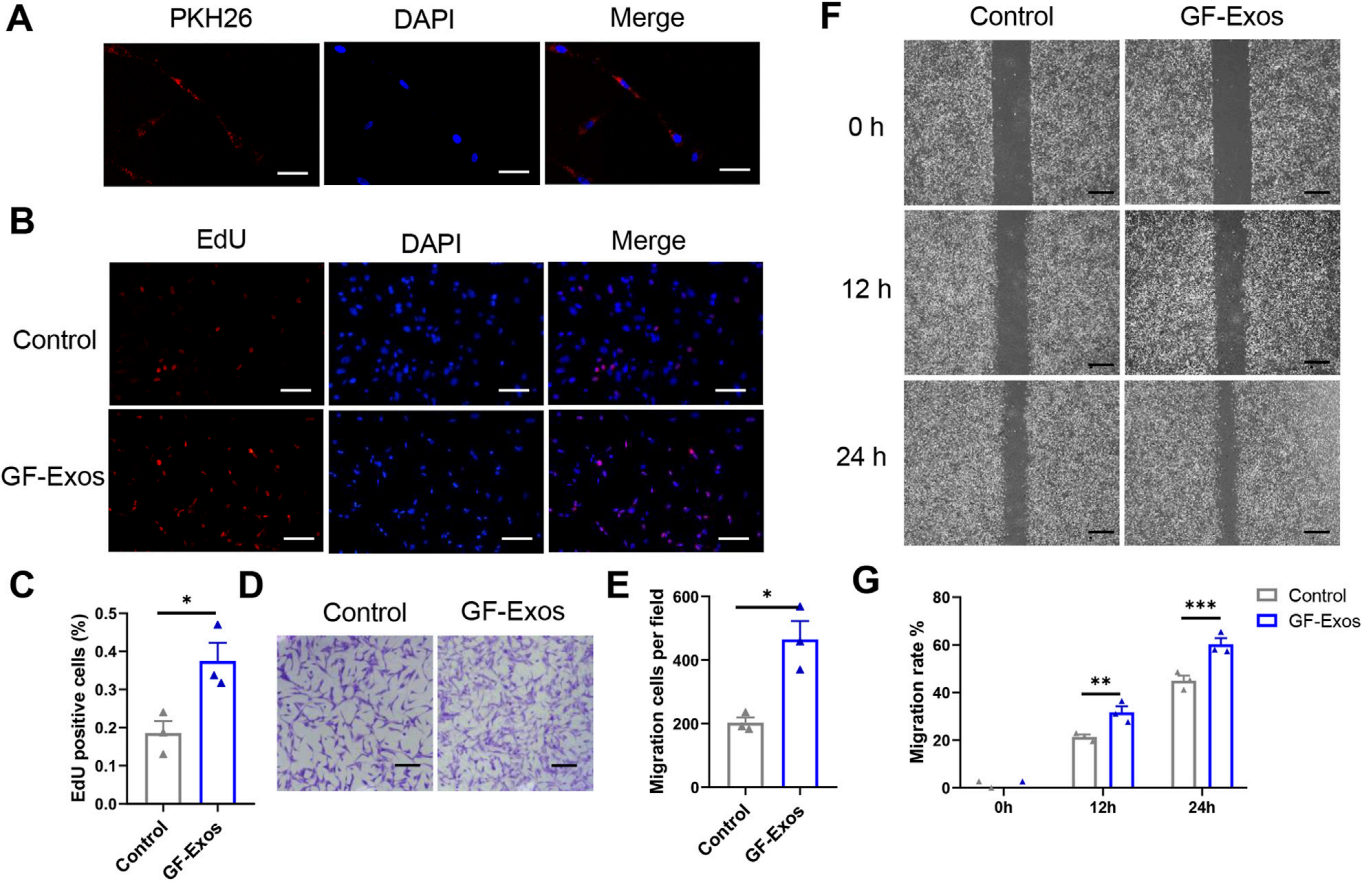
*In vitro* effect of GF-Exo on Tendon cells. **(A)** Uptake of PKH26-labeled GF-Exos by microglial cells *in vitro*. (Scale bar = 50 µm) **(B)** Proliferation of primary tendon cells treated with control or GF-Exos. The 5-ethynyl-2′-deoxyuridine (EdU)-positive cells represent new dividing cells and are stained (red). The nucleus was counterstained with 4′,6-diamidino-2-phenylindole (DAPI; blue). (Scale bar = 50 µm) **(C)** Quantification of Edu-positive cells in control and GF-Exos-treated groups. **(D)** Representative microscopic images of cells that migrated through the transwell in the migration assay (Giemsa stain). (Scale bar = 50 µm) **(E)** Quantification of migration cells per field in control and GF-Exos-treated groups. **(F)** Representative microscopic images of scratch closure in control and GF-Exos-treated groups at 0, 12, and 24 h (Scale bar = 200 µm) **(G)** Quantification of scratch closure in the different groups at 0, 12, and 24 h *p < 0.05, **p < 0.01, ***p < 0.001 vs. Control; n = 3; All data are shown as the mean ± Standard Error of the Mean (SEM); Statistical significance was determined by Student’s t-test.

To identify the internalization of GF-Exos, tendon cells was co-incubated with PHK26-labeled (red fluorescence) exosomes *in vitro* andlaser confocal microscopy revealed that tendon cells successfully internalized exosomes (Figure 3A). Subsequently, the EDU experiment demonstrated that GF-Exos treatment significantly enhanced the proliferation ability of tendon cells (Figures 3B,C).

In addition, we also explored the effect of GF-Exos on the migration ability of tendon cells. The staining images and quantitative analysis proved that the average number of migrating cells in the experimental group exceeded 400, significantly higher than 200 cells in the control group (Figures 3D,E). Similarly, the scratch experiment results showed that migration rate in control group was 21% and 45% at 12 and 24 h, respectively; while in the GF-Exos treatment group, the cell migration rate reached 31% and 66% at 12 and 24 h (Figures 3F,G). These results indicated that GF-Exos significantly enhanced the migration ability of tendon cells.

### 3.4 GF-Exos-mediated antioxidant activity and macrophage polarization

**FIGURE 4:**
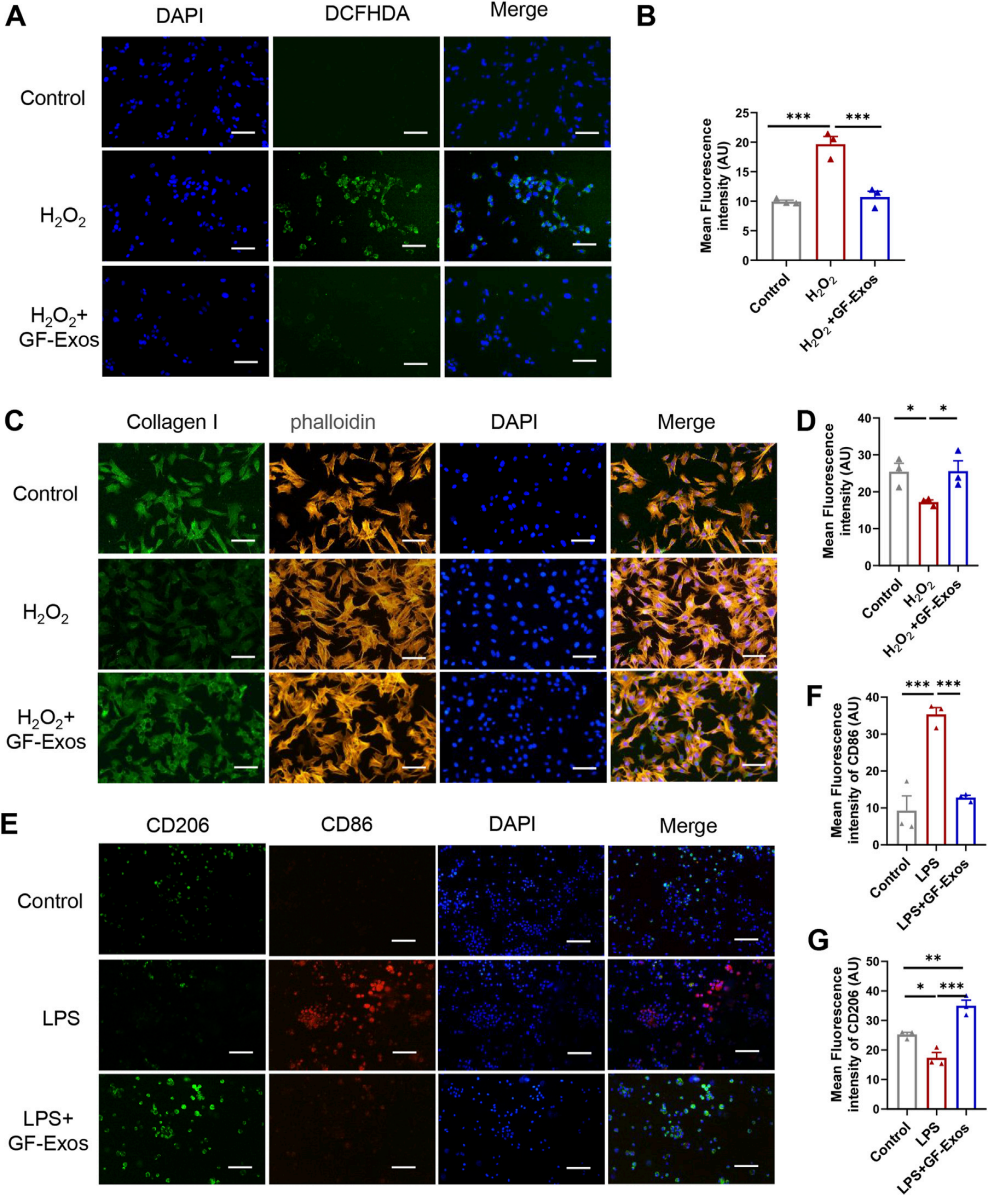
GF-Exos alleviates H_2_O_2_-induced oxidative stress and type I collagen synthesis disorder and regulates macrophage polarization. **(A)** ROS detection in primary tendon cells treated with Control and GF-Exos under H_2_O_2_ stimulation, where green fluorescence (DCFHDA) indicates ROS levels, with DAPI staining for nuclei. (Scale bars: 50 µm) **(B)** Quantification of mean fluorescence intensity of DCF in control, H_2_O_2_ and H_2_O_2_+GF-Exos groups. **(C)** Immunofluorescence staining of the primary tendon cells after being cocultured with control, and GF-Exos under H2O2 stimulation. Collagen I (green); phalloidin (Yellow); Nucleus (Blue). (Scale bars: 50 µm) **(D)** Quantification of mean fluorescence intensity of Collagen I in control, H_2_O_2_ and H_2_O_2_+GF-Exos groups. **(E)** Immunofluorescence staining of the mouse alveolar macrophages cells after being cocultured with control, and GF-Exos under LPS stimulation. CD206 (green); CD86 (Red); Nucleus (DAPI, Blue). (Scale bars: 50 µm) **(F)** Quantification of mean fluorescence intensity of CD86 in control, LPS and LPS + GF-Exos groups. **(G)** Quantification of mean fluorescence intensity of CD206 in control, LPS and LPS + GF-Exos groups. *p < 0.05, **p < 0.01, ***p < 0.001; n = 3; All data are shown as the mean ± Standard Error of the Mean (SEM); Statistical significance was determined by one-way ANOVA with Fisher’s LSD test.

Studies have shown that excessive production of reactive oxygen species (ROS) and oxidative stress are key factors in the pathogenesis of tendinitis (Kračun et al., 2025). Based on this, the use of natural source exosomes with ROS scavenging and anti-inflammatory properties may provide new therapeutic strategies for tendinitis. To investigate whether GF-Exos has a ROS-scavenging effect, an H_2_O_2_ assay was performed as previously reported (Im et al., 2023). The intracellular ROS levels were quantitatively evaluated using the ROS-sensitive fluorescent probe DCFHDA, which diffused into the cells and emitted fluorescence upon oxidation. The enhanced green fluorescence observed in tendon cells treated with H_2_O_2_ indicated an increase in ROS levels (Figures 4A,B). Notably, the application of GF-Exos significantly attenuated the ROS levels induced by H_2_O_2_, as evidenced by the decreased fluorescence intensity in the treated samples (Figures 4A,B). Additionally, we evaluated the regulatory effect of GF-Exos on type I collagen synthesis in tendon cells under oxidative stress conditions. We used immunofluorescence to detect the expression level of type I collagen. The results showed that the signal intensity of type I collagen in tendon cells treated with H_2_O_2_ was significantly lower compared to the control group; however, the co-treatment with GF-Exos effectively restored the expression level of type I collagen (Figures 4C,D). These results indicated that GF-Exos had a protective effect on tendon cells under oxidative stress conditions and may maintain ECM homeostasis by regulating the synthesis of type I collagen.

The evidence from animal models indicates that the enhanced activity of M2 macrophages leads to reduced scar formation, accelerated healing, decreased inflammation and enhanced biomechanical strength, making M2 polarization a promising therapeutic strategy for tendinitis (Sunwoo et al., 2020). To investigate the potential regulatory effects of GF-Exos on macrophage polarization, we incubated GF-Exos with mouse alveolar macrophages stimulated by lipopolysaccharide (LPS). Subsequently, CD86 and CD206 immunofluorescence was performed. The experimental results showed that the expression of CD86, a marker of M1 macrophages (pro-inflammatory macrophages), was upregulated in the LPS-treated group compared to the control group (Figures 4E,F). The expression of CD86 significantly decreased after co-treatment with GF-Exos, which was comparable to the level of the untreated group (Figures 4E,F). The expression level of CD206, a marker of M2 macrophages, was significantly lower in LPS group than that of the control group (Figures 4E,G). By contrast, co-treatment with GF-Exos significantly increased the signal intensity of CD206 compared to the control group (Figures 4E,G). These findings implied that GF-Exos could induce M2 polarization of macrophages under inflammatory conditions.

### 3.5 Transcriptomic analysis of GF-Exos-mediated protection against H_2_O_2_-Induced oxidative stress in tendon cells

**FIGURE 5:**
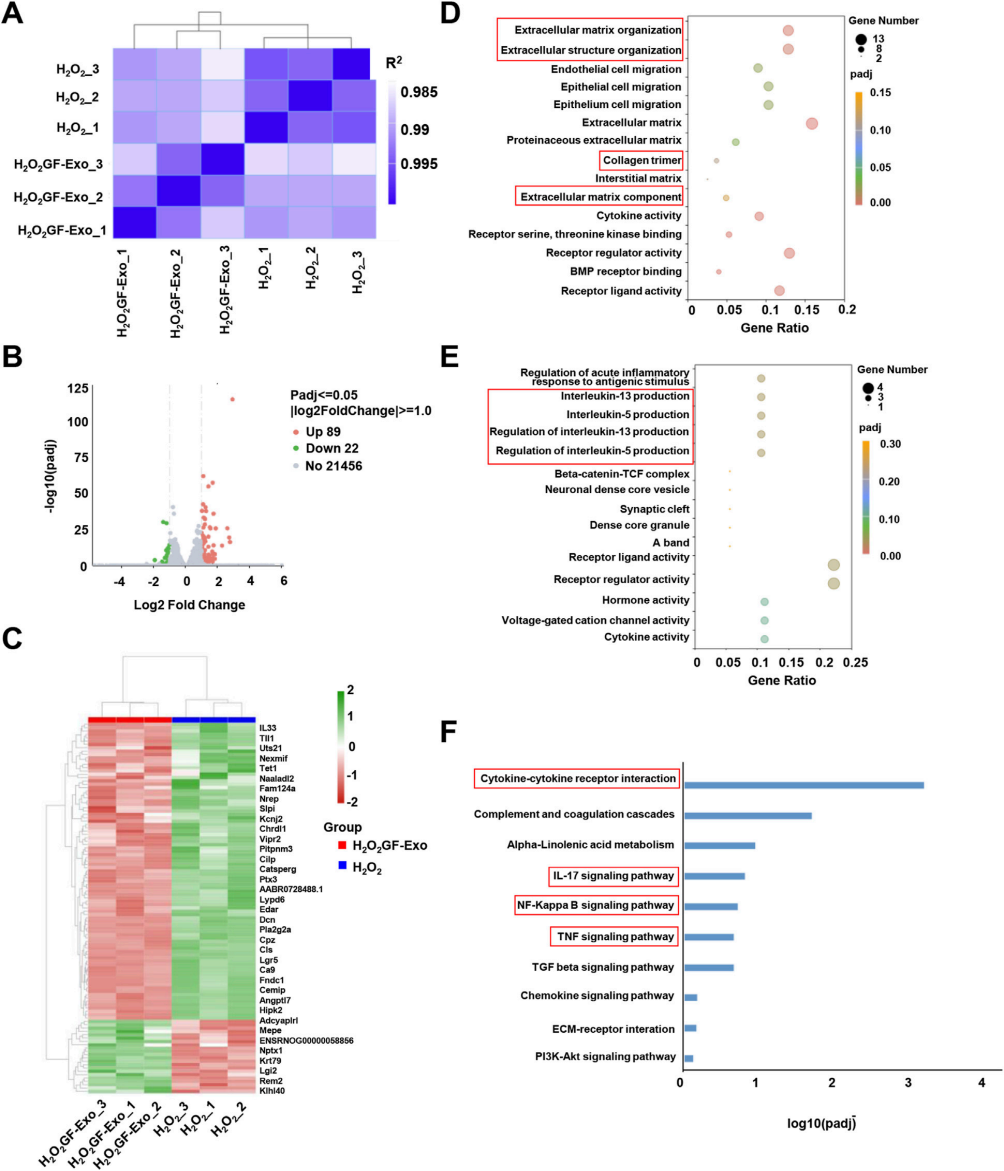
Changes in transcriptome profile of GF-Exo-treated tendon cells against ROS *in vitro*. **(A)** Pearson correlation between H_2_O_2_ and H_2_O_2_+GF-Exos RNA-Seq data. The change of the square (*R* ^2^) value of the correlation coefficient of Pearson is indicated by the change of the blue color. The deeper color indicates a bigger *R* ^2^ value and a higher correlation between samples. **(B)** The volcano plot of differentially expressed genes (DEGs) between H_2_O_2_+GF-Exos group and H_2_O_2_ group. **(C)** Hierarchical clustering of the differentially expressed genes. The blue bands represent downregulated genes, and the red bands represent upregulated genes. **(D)** The results of Gene Ontology (GO) enrichment analysis in upregulated genes. **(E)** The results of Gene Ontology (GO) enrichment analysis in downregulated genes. **(F)** Enrichment analysis of the KEGG pathways for the differentially expressed genes.

To elucidate the molecular mechanisms underlying the ROS-protective effects of GF-Exos on tendon cells *in vitro*, we conducted transcriptomic profiling through RNA sequencing (RNA-seq) analysis. Figure 5A showed the results of the correlation analysis between samples. The individual biological replicates of the H_2_O_2_ treatment group clustered together, and the three biological replicates of the GF-Exos treatment group clustered together (Figure 5A). The R2 values between the biological replicate samples of the two treatment groups were all greater than 0.92 (Figure 5A). Volcano plot was plotted for identifying differentially expressed genes (Figure 5B). The heatmap detailed the gene expression levels in the samples, and hierarchical clustering represented different expression profiles as shown in the legend (Figure 5C).

To conduct a more in-depth study on the affected biological functions and pathways, we performed Gene Ontology (GO) enrichment analysis and Kyoto Encyclopedia of Genes and Genomes (KEGG) pathway enrichment analysis. The GO analysis emphasized that the genes significantly upregulated in the GF-Exos treatment group involved in the activation of cell migration and tissue repair and regeneration signaling pathways (Figure 5D). Notably, the upregulation of ‘Extracellular matrix\structure organization’, ‘Extracellular matrix component’ and ‘Collagen trimer’ genes indicated that GF-Exos might improve the tendon structure disruption induced by H_2_O_2_ through enhancing collagen fiber cross-linking and ECM stability (Figure 5D). The genes significantly downregulated by GF-Exos treatment were concentrated in inflammation and stress-related pathways (Figure 5E). For example, the inhibition of genes related to IL-5/IL-13 generation regulation suggested that GF-Exos treatment might alleviate tendon inflammation by reducing pro-inflammatory cytokine release (Figure 5E). These findings highlighted the dual effects of GF-Exos on antioxidation, anti-inflammation, and ECM repair, demonstrating its potential in the treatment of tendinopathy. The KEGG pathway enrichment analysis showed a significant modulation of cytokine-cytokine receptor interaction in tendon cells with GF-Exos treatment post H_2_O_2_ stimulation (Figure 5F). This pathway mediates cytokine signal transduction and regulates inflammatory/immune responses (Spangler et al., 2015). Additionally, inflammatory pathways such as IL-17, NF-κB and TNF were also modulated (Figure 5F), implying that GF-Exos alleviated H_2_O_2_-induced oxidative stress damage in tendon cells by regulating the dynamic balance of pro-inflammatory and anti-inflammatory signals.

**FIGURE 6:**
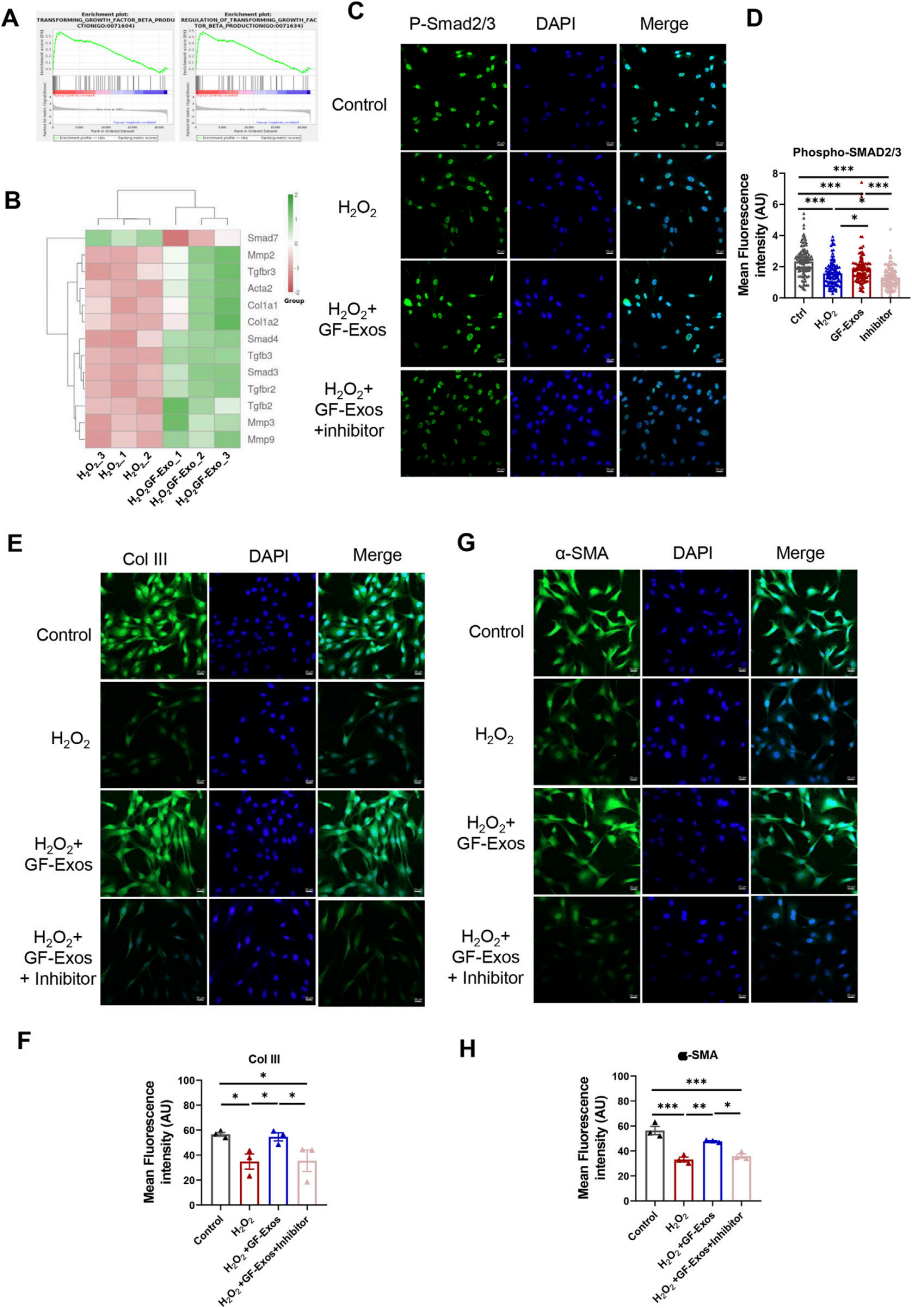
GF-Exos alleviates H_2_O_2_-induced oxidative stress through TGF-β signaling. **(A)** GSEA plot of the genes associated with transforming growth factor beta production and regulation of transforming growth factor beta transforming. **(B)** Expression profiles of genes related to TGF-β signaling. **(C)** Representative images of immunofluorescence staining of phospho-smad2/3 in control, H_2_O_2_, , H_2_O_2_+ GF-Exos and H_2_O_2_+ GF-Exos + TGF-β inhibitor groups. phospho-smad2/3 (green); Nucleus (DAPI, Blue). (Scale bars: 20 µm). **(D)** Quantification of mean fluorescence intensity of phospho-smad2/3 in control, H_2_O_2_, H_2_O_2_+GF-Exos, and H_2_O_2_+GF-Exos + TGF-β inhibitor groups. The signals from more than 100 cells per groups were quantified. **(E)** Representative images of immunofluorescence staining of collagen III in control, H_2_O_2_, H_2_O_2_+ GF-Exos and H_2_O_2_+ GF-Exos + TGF-β inhibitor groups. Collagen III (green); Nucleus (Blue). (Scale bars: 20 µm) **(F)** Quantification of mean fluorescence intensity of collagen III in control, H_2_O_2_, H_2_O_2_+ GF-Exos and H_2_O_2_+ GF-Exos + TGF-β inhibitor groups. **(G)** Representative images of immunofluorescence staining of α-SMA in control, H_2_O_2_, H_2_O_2_+ GF-Exos and H_2_O_2_+ GF-Exos + TGF-β inhibitor groups. α-SMA (green); Nucleus (Blue). (Scale bars: 20 µm) **(H)** Quantification of mean fluorescence intensity of α-SMA in control, H_2_O_2_, H_2_O_2_+ GF-Exos and H_2_O_2_+ GF-Exos + TGF-β inhibitor groups. p < 0.05, **p < 0.01, ***p < 0.001; n = 3 **(E,F)**; All data are shown as the mean ± Standard Error of the Mean (SEM); Statistical significance was determined by one-way ANOVA with Fisher’s LSD test.

Additionally, KEGG analysis indicated significant regulation of TGF-β signaling in H_2_O_2_+GF-Exos groups (Figure 5F), while GSGA highlighted the relationship between TGF-β signaling and GF-Exos-mediated protection (Figure 6A). Subsequent analysis of TGF-β-related genes revealed that *Smad7*—a negative regulator of TGF-β signaling (Yan et al., 2009) —was downregulated in the H_2_O_2_+GF-Exos group. Conversely, expression of the primary TGF-β signal transducers *Smad3* and *Smad4* increased following GF-Exos treatment (Figure 6B). Furthermore, downstream TGF-β target genes (*ACTA2*, *COL1A1*, and *COL1A2*) were upregulated in H_2_O_2_+GF-Exos groups (Figure 6B). These findings collectively indicated that GF-Exos protected tendon cells from H_2_O_2_-induced oxidative stress through TGF-β signaling pathway activation. Similar results were confirmed by immunofluorescence staining of p-Smad2/3 (Figures 6C,D). H_2_O_2_ treatment significantly decreased phosphorylated Smad2/3 levels compared to controls, while GF-Exos treatment reversed this downregulation (Figures 6C,D). TGF-β inhibitors partially attenuated the protective effects of GF-Exos (Figures 6C,D). The expression levels of TGF-β target genes in the H_2_O_2_+GF-Exos group were restored to control levels relative to the H_2_O_2_ group (Figures 4C,D; Figures 6E–H).

Collectively, these transcriptomic and functional analyses demonstrate that GF-Exos protect tendon cells from H_2_O_2_-induced oxidative stress through multifaceted mechanisms, including ECM restoration, suppression of inflammatory pathways, and specific activation of the TGF-β/Smad signaling cascade.

### 3.6 The *in vivo* therapeutic effect and efficacy of MN@GF-Exos patches

**FIGURE 7:**
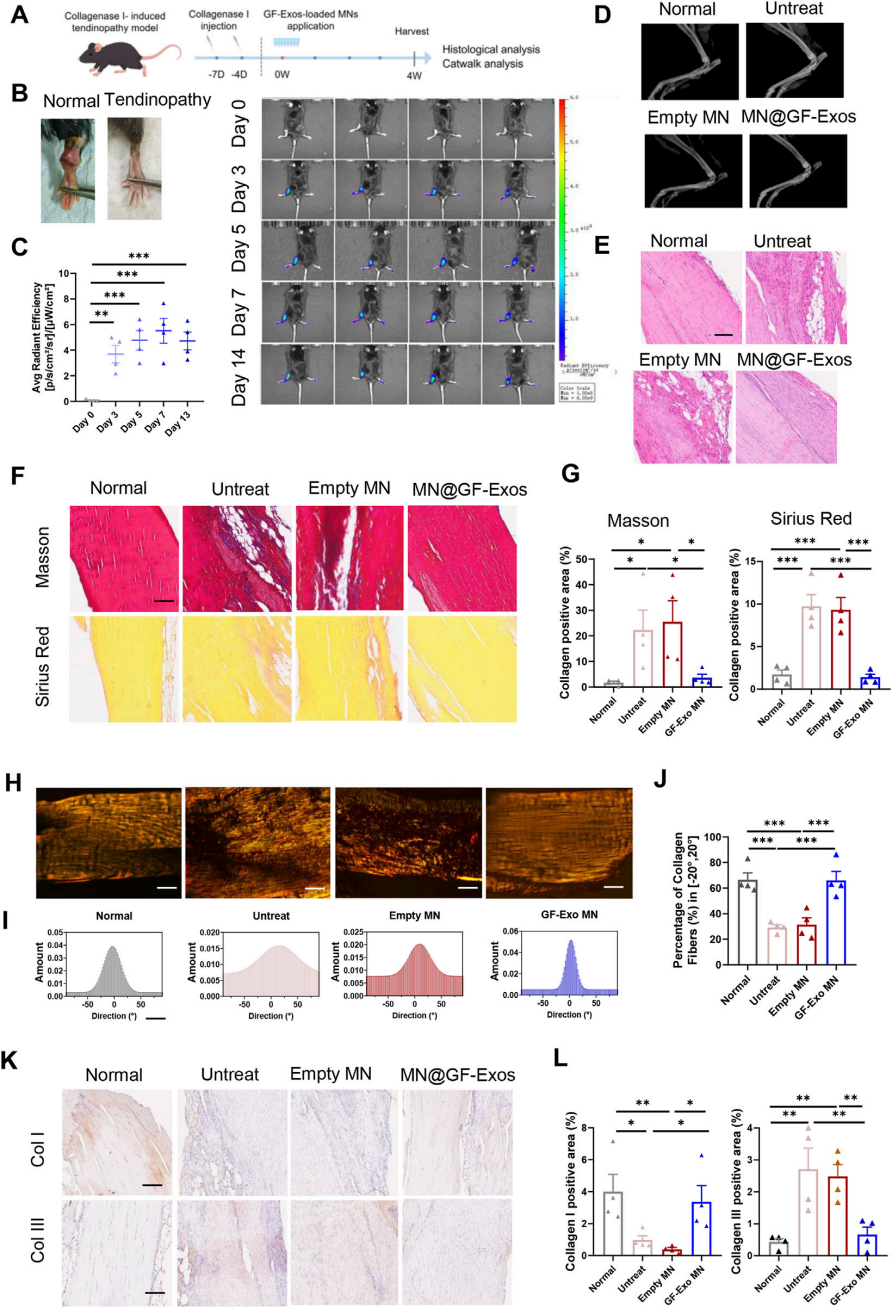
Local administration of MN@GF-Exos efficiently delays the progression of collagenase l-induced tendinopathies in mice. **(A)** Scheme of experiment design (By Figdraw). **(B)** Gross view of skin contours of tendons in the fourth week postoperatively. **(C)** *In vivo* imaging of mice after application of DiD-labeled GF-Exos MN at tendon site. Comparison of average radiance [p/s/cm2/sr] between different groups was measured at Day 0, 3, 5, 7, and 14 after GF-Exos MN administration. **(D)** Micro-CT scans of repaired Achilles tendons of each group in the fourth week postoperatively. **(E)** HE staining of tendons from different groups in the fourth week postoperatively. (Scale bar = 100 µm) **(F)** Sirius red and Masson’s trichrome staining of tendons from different groups in the fourth week postoperatively. (Scale bar = 100 µm) **(G)** Quantification analysis of Sirius red and Masson’s trichrome staining. **(H)** Representative images of Sirius red staining of tendons from different groups. **(I)** Representative results of collagen orientation analysis of tendon tissue from different group according to Pseudocolor images. **(J)** Quantification of percentage of collagen fibers with orientation between −20 to 20°. **(K)** Immunohistochemical staining of COL1 and COL3 of sections of each group in the fourth week postoperatively. **(L)** Quantification of COL1 and COL3 expression in different groups. *p < 0.05; **p < 0.01; ***p < 0.001; n = 4 (C to L); All data are shown as the mean ± Standard Error of the Mean (SEM). Statistical significance was determined by one-way ANOVA with Fisher’s LSD test.

To evaluate the efficacy of MN@GF-Exos treatment for tendinopathy *in vivo*, a type I collagenase-induced mouse Achilles tendonitis model was established (Figures 7A,B). The *in vivo* imaging of DiD-labelled GF-Exos at the mouse tendon site was able to detect the signals for at least 14 days post-application (Figure 7C). Four weeks after treatment, X-ray examination showed that there was no heterotopic ossification in all groups, confirming the safety of the treatment (Figure 7D). Histological analysis of the Achilles tendon tissue was conducted to gain a deeper understanding of the therapeutic effect. H&E staining showed that compared with other groups, MN@GF-Exos significantly reduced the formation of vacuolar-like structures and promoted the recovery of aligned collagen structures (Figure 7E). Masson’s trichrome and Sirius red staining revealed a significant increase in fibrotic deposition within untreated group and empty MN-treated groups compared to normal controls (Figures 7F,G). In contrast, MN@GF-Exos intervention restored collagen-positive areas to levels comparable to healthy tissue (Figures 7F,G). Furthermore, collagen fiber alignment was quantified by measuring angular distribution relative to the tendon’s longitudinal axis (Figures 7H–J). Approximately 30% of collagen fibers aligned within ±20° in the untreated and empty MN groups, whereas over 60% aligned within ±20° in treatment groups, closely resembling the healthy control group (Figures 7H–J). The highly aligned fibers in treatment groups indicated superior mechanical strength and better tendon-tissue-repair outcomes than the disorganized fibers observed in untreated and empty MN groups. Quantitative immunohistochemical analysis demonstrated collagen I deposition and collagen III levels in the tendinitis mice was respectively significantly downregulated and upregulated compared to normal groups (Figures 7K,L). MN@GF-Exos treatment restored collagen I and III levels which is closely approximating physiological levels observed in healthy tendons, indicating restored extracellular matrix homeostasis (Figures 7K,L).

**FIGURE 8:**
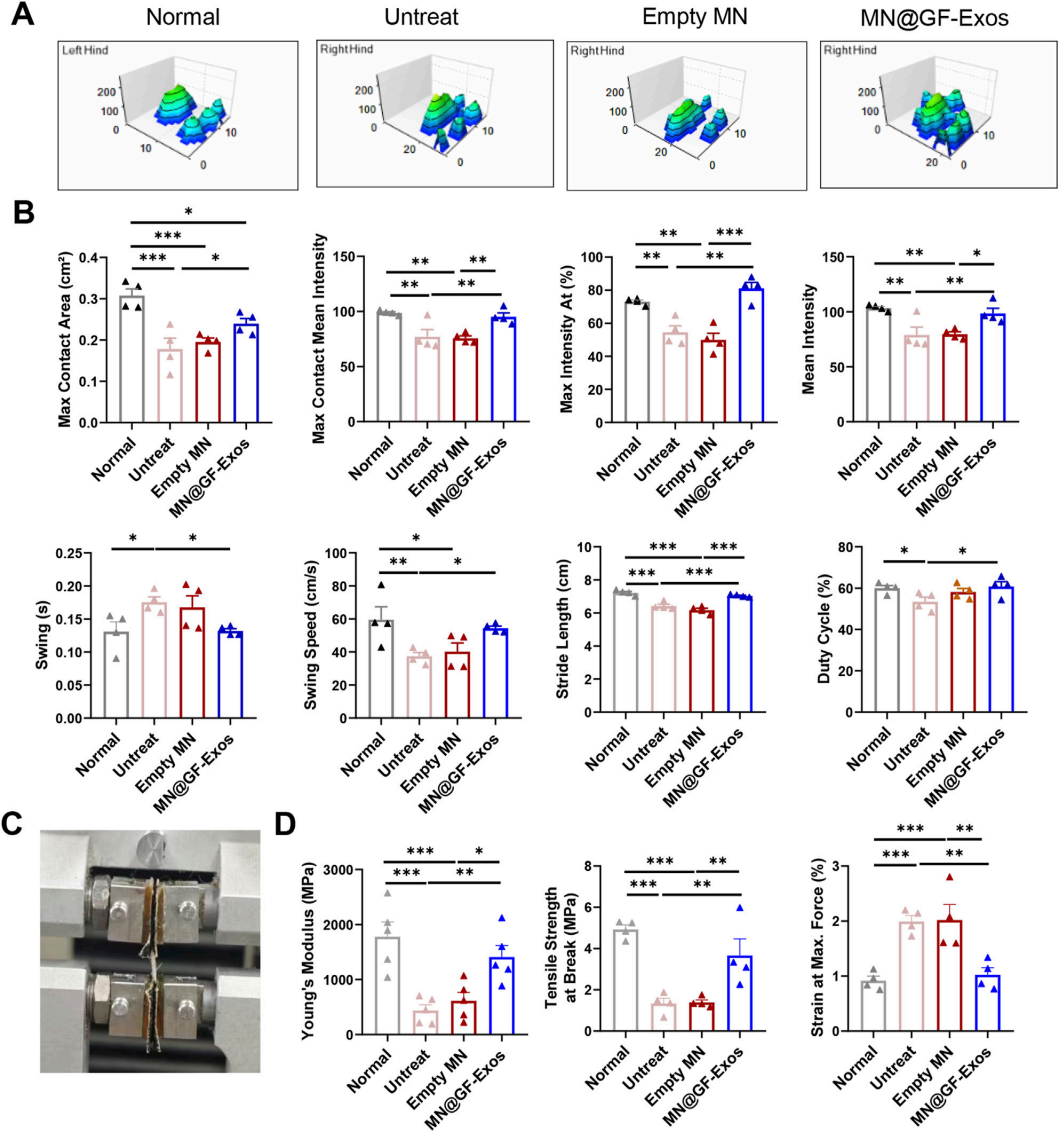
Catwalk for gait analysis. **(A)** Representative 3D footprint intensity images of the left (control) and right hind leg (tendinitis) in the various experimental groups at 4 weeks after treatment. **(B)** The quantification analysis of catwalk for gait analysis. **(C)** Mechanical testing of the Achilles tendon. **(D)** Comparison of the Young’s Modulus, Tensile strength at Break, and Strain at Max. Force of the Achilles tendon in different group. *p < 0.05, **p < 0.01, ***p < 0.001; n = 4; All data are shown as the mean ± Standard Error of the Mean (SEM). Statistical significance was determined by one-way ANOVA with Fisher’s LSD test.

We also evaluated the motor ability of mice using the CatWalk system. The 3D footprint intensity maps of the affected hind limb (right hind) and the control hind limb (left hind) were captured (Figure 8A) and quantitatively evaluated (Figure 8B). Compared with the untreated group and the blank microneedle treatment group, the mice treated with MN@GF-Exos exhibited better gait patterns and were closer to normal mice. These results indicated that MN@GF-Exos improved the walking performance of mice. Mechanical testing of tendons revealed that key parameters—including Young’s modulus, tensile strength at break, and strain at maximum force—in the treatment group approached those of native tendons, whereas values in the untreated and empty MN groups remained significantly lower or higher (Figures 8C,D). These results demonstrate superior mechanical properties in the treatment group compared to untreated and empty MN controls.

In summary, grapefruit-derived exosome-loaded microneedle therapy synergistically modulated collagen metabolism by promoting type I collagen synthesis while suppressing type III deposition. This approach concurrently inhibited fibrotic progression and restored tendon structural integrity and biomechanical function. Notably, the intervention presents no risk of pathological ossification, offering a promising translational strategy for the clinical management of chronic tendinopathy.

### 3.7 *In vivo* biological safety test

**FIGURE 9:**
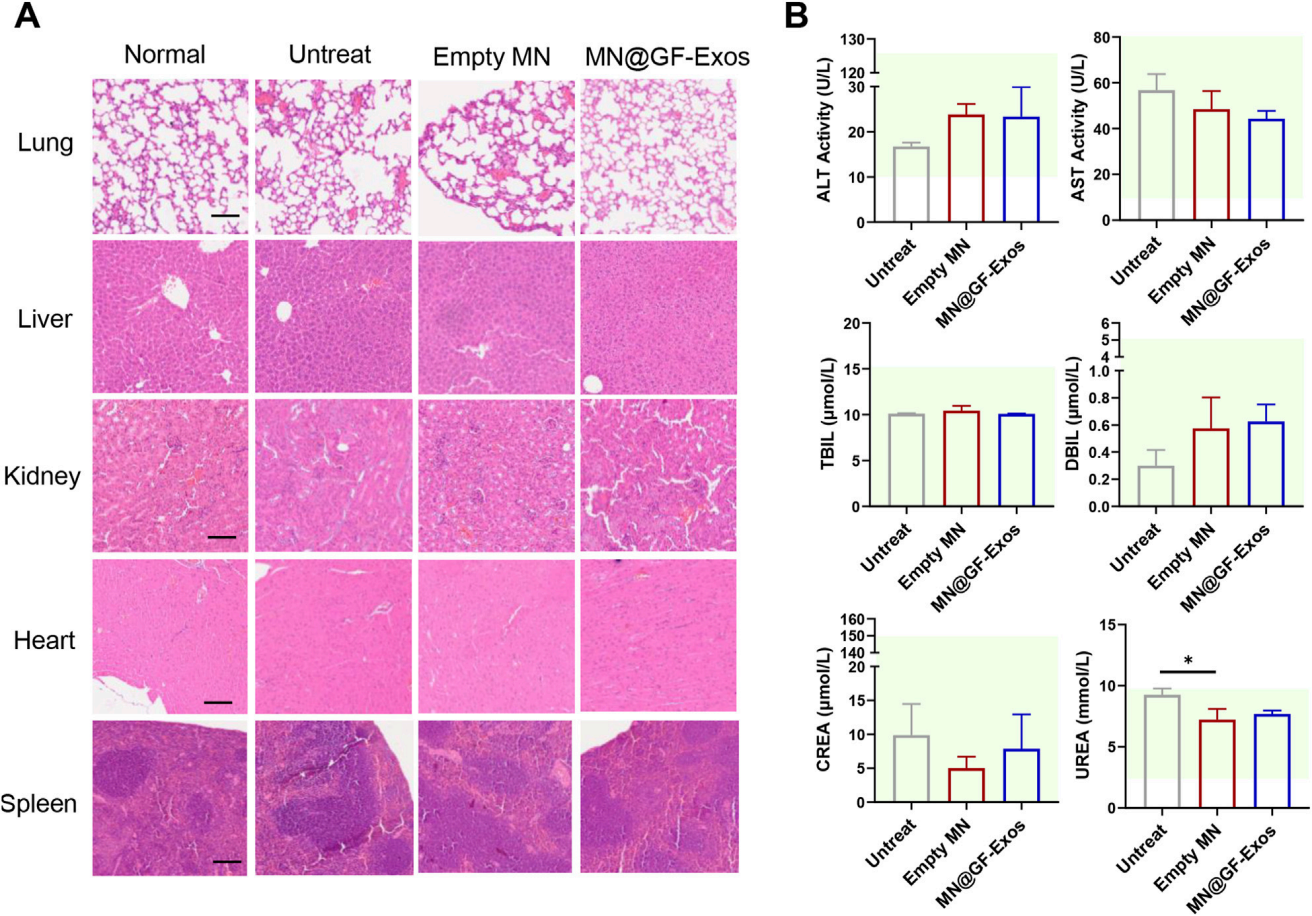
Evaluation of Biosafety of GF-Exo MN. **(A)** H&E-stained sections of rat heart, kidney, liver, lung, and spleen from normal, untreat, empty MN, and MN@GF-Exos groups after a 4-week intervention. (Scale bar = 100 µm) **(B)** Biochemical analysis of serum markers indicating organ function in mice with various interventions. The panels display levels of ALT, AST, TBIL, DBIL, CREA, and UREA. The green-shaded area indicates the established reference range for healthy control mice in this experiment. *p < 0.05; **p < 0.01; ***p < 0.001; n = 4; All data are shown as the mean ± Standard Error of the Mean (SEM). Statistical significance was determined by one-way ANOVA with Fisher’s LSD test.

An optimal therapeutic system must balance dual imperatives: achieving targeted therapeutic efficacy while maintaining biocompatibility standards. H&E staining of major organs (heart, liver, spleen, lung, kidney) from MN@GF-Exos-treated mice revealed preserved tissue architecture without degenerative lesions or inflammatory infiltration, validating the biosafety profile of the microneedle-mediated grapefruit exosome delivery platform with minimal systemic toxicity and absence of immunogenic responses (Figure 9A). In addition, serum biochemical indicators showed that there was no significant difference between MN@GF-Exos and the untreated group (Figure 9B). And all assessed parameters fall within reference range for healthy control mice (Figure 9B). Therefore, MN@GF-Exos demonstrated biological safety, and tissue and biochemical tests confirmed that they had no toxicological effects on the main organ functions.

## 4 Discussion

The pharmacological treatment of tendinopathies remains challenging due to the limited vascularization of tendons, which results in low drug availability at the target site (Loiacono et al., 2019). Here, we innovatively combined GF-Exos with the microneedle (MN) delivery system for local therapeutic interventions of tendinopathy. MN@GF-Exos demonstrated dual therapeutic mechanisms *in vitro*: pro-proliferative, anti-oxidative, and collagen-promoting effects on tendon cells under oxidative stress, and M2 macrophage-polarizing effects to ameliorate the inflammatory microenvironment. The *in vivo* application of MN@GF-Exos in tendinitis models not only reduced structural damage but also restored biomechanical function, as reflected in normalized gait parameters. Furthermore, systematic safety assessments confirmed their clinical translatability, supporting MN@GF-Exos as a multi-mechanistic therapeutic platform.

The therapeutic potential of MN@GF-Exos is particularly relevant to chronic tendinopathies, which are exacerbated by accelerated tendon aging (Kwan et al., 2023). Notably, aged tendons exhibit impaired healing responses to conventional therapies targeting pain relief or surgical repair, underscoring the need for mechanistically informed treatments (Morya et al., 2024). The aging process is linked to chronic oxidative stress resulting from elevated reactive oxygen species (ROS) levels (Liguori et al., 2018; Duan et al., 2023). Senescent tenocytes exhibit impaired migration, contributing to poor tendon healing (Wang et al., 2023). Tendon homeostasis depends on tenogenesis and ECM production (Hou et al., 2021). Cellular senescence reduces tenocyte numbers and protein synthesis, ultimately disrupting collagen/ECM organization (Tsai et al., 2011; Birch et al., 2016). Given the multifactorial nature of tendon aging (oxidative stress, cellular senescence, and ECM dysregulation), multi-targeted therapeutic strategies are urgently required to address these interconnected pathological processes.

To address these challenges, exosome-based therapies, leveraging their inherent multifactorial cargo, have emerged as a promising strategy to simultaneously target oxidative stress, cellular senescence, and ECM dysregulation (Garaeva et al., 2021; Robbins and Morelli, 2014; Rayner and Hennessy, 2013; Mu et al., 2014; Aswani et al., 2024). The GF-Exos that we focus on in this study have been proved to modulate receptor cell functions by regulating inflammatory responses, eliminating reactive oxygen species (ROS), and promoting cell proliferation and migration following cellular uptake (Wang et al., 2014; Savcı et al., 2021; Mu et al., 2014), which was also verified in our study. Such multi-targeted function provides a theoretical basis for the application of GF-Exos in tendon repair. In addition, the synergistic action of GF-Exos should be attributed to its composite cargos: Quantitative proteomics previously identified that GF-Exos were enriched with naringenin (a potent anti-inflammatory/antioxidant) and carry proteins regulating carbohydrate/lipid metabolism (Mu et al., 2014).

Further mechanistic investigations revealed that GF-Exos may promote tendon repair by modulating TGF-β pathway activation. Previous studies have demonstrated that TGF-β signaling participates in tendon cell proliferation and migration, collagen synthesis, and matrix production/metabolism following tendon injury (Li et al., 2022). Transcriptomic and immunofluorescence analyses of tendon cells indicated that TGF-β signaling was activated in H_2_O_2_+GF-Exos compared to H_2_O_2_ group. This H_2_O_2_-related inhibition on TGF-β signaling may be attributed to H_2_O_2_-induced cysteine sulfenylation of Smad2, which subsequently reduced Smad2 phosphorylation (Huang et al., 2021). GF-Exos partially rescued both the phosphorylation level of Smad2 and the expression of α-SMA and collagen III—established downstream targets of TGF-β signaling (Li et al., 2022; Fakatava et al., 2023).

GF-Exos were loaded into GelMA MNs for *in vivo* application. Crucially, exposure of GF-Exos to 30°C during the encapsulation process did not induce detectable alterations in vesicle morphology and mean size, confirming the thermostability of exosomes’ architecture under manufacturing conditions. This structural preservation correlated with functional integrity, as evidenced by the therapeutic efficacy of MN@GF-Exos *in vivo*, and was consistent with previous reports demonstrating bioactivity retention in exosomes after being loaded into microneedles (Yuan et al., 2022; Liu et al., 2021; Zhang et al., 2024).

The functional recovery observed in MN@GF-Exos group was strongly supported by gait metrics (Figure 7B). The partially restored maximum contact area and near-normal stride length indicated effective tendon regeneration, allowing proper weight-bearing distribution. Notably, the significantly shortened swing in MN@GF-Exos group (0.13 s vs. Untreat 0.18 s) suggested reduced compensatory limb retraction, likely due to diminished pain responses as evidenced by duty cycle normalization. The improvements in gait parameters of MN@GF-Exos group implied that GF-Exos not only promoted structural repair but also restored neuromuscular coordination, a critical aspect often overlooked in tendon regeneration studies.

Traditional treatments for tendinitis, including nonsteroidal anti-inflammatory drugs, corticosteroids, and physical therapy, can provide symptom relief but face important drawbacks. These conventional approaches work through narrow mechanisms - primarily reducing inflammation - without supporting tissue repair, and may even negatively affect the regeneration of tissue (Loiacono et al., 2019; Andres and Murrell, 2008). In contrast, MN@GF-Exos overcomes these limitations through a dual synergistic mechanism. First, it significantly enhances tendon cell viability under oxidative stress and promotes collagen synthesis. Second, the multiple components carried by GF-Exos induce macrophage M2 polarization, thereby improving the inflammatory microenvironment. This combined approach of promoting repair while controlling inflammation offers distinct advantages over traditional single-mechanism treatments.

Current *in vitro* evaluation systems for biomimetic biomaterials and pharmaceutical agents targeting musculoskeletal disorders such as tendinopathy primarily rely on traditional two-dimensional (2D) cell co-culture models. However, the simplicity of these systems fails to fully recapitulate the heterogeneity and complexity of *in vivo* environments, necessitating experimental studies and *in vivo* testing in animal models (Urzì et al., 2023). To enhance the consistency between *in vitro* and *in vivo* material assessments, future research should prioritize the adoption of biomimetic dynamic testing platforms, such as three-dimensional (3D) mechano-transduction systems equipped with real-time mechanical loading capabilities (Im et al., 2025). These advanced platforms could simulate multi-cellular co-culture, dynamic ECM remodeling, and mechanobiological coupling feedback, thus enabling more accurate prediction of material functionality *in vivo* and accelerating the clinical translation of therapeutic strategies.

In conclusion, our comprehensive studies affirm that MN@GF-Exos serve as a potent intervention for tendinitis. Its therapeutic efficacy is supported by histological and Catwalk of gait analysis the showed reduced tendon damage and enhanced tendon function. Further studies are needed to verify their long-term efficacy through large animal experiments and to further explore their molecular mechanism to accelerate the translation of this nanotherapy system to clinical application.

## Data Availability

The datasets presented in this study can be found in online repositories. The names of the repository/repositories and accession number(s) can be found in the article/Supplementary Material.
