# Supplementary material for: Restoration of tendon repair microenvironment by grapefruit exosome-loaded microneedle system for tendinopathy therapy
Source: Front Bioeng Biotechnol. 2025 Jul 28;13:1615650. doi: 10.3389/fbioe.2025.1615650 (PMC12336195; doi:10.3389/fbioe.2025.1615650)
Supplement: Supplementary file 1 [file Supplementaryfile1.docx]

Supplementary Material

## Cell Counting Kit (CCK)-8 assay

The cell viability after GF-Exos treatment was measured by the CCK-8 reagent (HY-K0301, MedChemExpress) according to the manufacturer’s protocol. In brief, tendon cells were seeded in 96-well plates at a density of 5×10³ cells/well and incubated with indicated concentration of GF-Exos. After treatment, 10 μL of CCK-8 reagent was added to each well. 450 nm OD values were determined with a microplate reader after 1 h of incubation at 37°C.


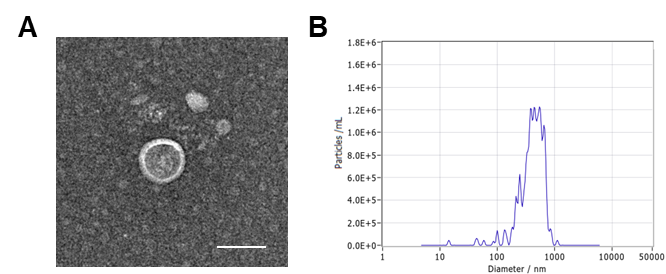


**Supplementary Figure 1.** Characterization of GF-Exos after 30 ℃ heating treatment**. (A)** Transmission electron microscopy (TEM) images depicting GF-Exos after two cycle of 30 ℃ incubation (50 minuts per cycle). (Scale bar = 500 µm) **(B)** Particle size of GF-Exos in (A) determined by Nanoparticle tracking analysis (NTA).\

**Supplementary Figure 2.** CCK8 test on tendon cell treated with different concentration of GF-Exos.
